# Enhanced Fire-Extinguishing Performance and Synergy Mechanism of HM/DAP Composite Dry Powder

**DOI:** 10.3390/ma18030533

**Published:** 2025-01-24

**Authors:** Lijing Wang, Shaowen Du, Zhiji Zhou, Yibo Guo, Qi Yang, Sai Yao, Haijun Zhang

**Affiliations:** School of Safety Science and Engineering, Key Laboratory of Civil Aviation Thermal Hazards Prevention and Emergency Response, Civil Aviation University of China, Tianjin 300300, China; ljwang@cauc.edu.cn (L.W.); dsw18010698197@163.com (S.D.); zj_zhou0520@163.com (Z.Z.); ybguo@cauc.edu.cn (Y.G.); q-yang@cauc.edu.cn (Q.Y.)

**Keywords:** hydromagnesite, composite dry powder, extinguishing agent, liquid fire, solid fire

## Abstract

Phosphate resources are non-renewable and are increasingly depleting. Currently, the primary raw material for commercial ABC dry powder fire-extinguishing agents is a processed product derived from the limited reserves of phosphorus ore. Consequently, there is an urgent imperative to innovate and develop novel types of dry powder fire-extinguishing agents. In this work, a simple physical blending process was utilized to modify the abundant and cost-effective hydromagnesite (HM) powder, which has been proven to be a promising dry powder extinguishant with a pronounced physical cooling effect on fire suppression. The HM powder added to 10 wt.% diammonium hydrogen phosphate (DAP) showed a shorter extinguishing time and a lower agent dosage compared with the unmodified powder and commercially available monoammonium phosphate (MAP) in both the liquid and solid fire experiments. Notably, the HM/DAP composite dry powder extinguished fires with the lowest CO concentration, indicating superior environmental friendliness and human safety. These findings indicate the potential of the HM/DAP composite dry powder as a promising candidate for future fire-extinguishing applications.

## 1. Introduction

The diminishing phosphorite resources present a formidable challenge to global resource security. Phosphorus resources are exceedingly scarce, constituting a non-metallic mineral resource with limited regenerative potential [1,2,3]. Global phosphorus reserves are estimated at approximately 70 billion tons [4,5]. High-grade phosphate rock is extracted from deposits that have formed over millions of years and are presently experiencing gradual depletion. Hence, the sustainable development and recycling of phosphorus resources are critical imperatives for the global community [6,7,8].

Currently, phosphate-based powders are utilized for extinguishing ABC fires in the fire protection systems in high-rise buildings, libraries, laboratories, electrical equipment rooms, airport tarmacs, and various other locations. These powders are produced using raw materials derived from the limited reserves of phosphate ore processing. There is an urgent need to develop novel, highly efficient dry powder fire-extinguishing agents with abundant reserves. Scholars have extensively explored various aspects of new agent development, including novel materials [9], structural adjustments such as microencapsulation [10,11], nanosizing [12], functionalization [13,14], and composition mixing regulation [15,16]. For instance, machine learning methods have been employed to investigate the fire performance of individual materials, with a focus on predicting the time to ignition and the peak heat release rate of polyvinylpyrrolidone (PVP) multilayer materials, thus advancing the development of fire-resistant materials [17]. Guo et al., utilized reactions involving urea, potassium, sodium acid carbonates, carbonates, or hydroxides to produce Monnex dry powder [18]. While these powders demonstrate effective fire-suppression capabilities, their current limitations include a high cost and a low bulk density, restricting them to laboratory research stages. Zhou et al. demonstrated that KAl(OH)_2_CO_3_ outperformed conventional BC dry powder agents, achieving suppression of n-heptane and aviation kerosene pool fires within 6.00 s and 7.21 s, respectively [19]. Jiang et al. optimized the hydrophobic silica encapsulation ratio of sodium bicarbonate, significantly enhancing the flowability and hydrophobic properties of ultrafine dry powder fire-extinguishing agents [12]. The dawsonite/superfine ABC dry powder fire-extinguishing agent (DW-SDP) developed by Zhou et al. [16] integrates the advantages of its individual components, enabling both chemical fire extinguishing and physical coverage. This dual mechanism results in an extinguishing time that is 47.4% faster than that of commercial ABC dry powder.

Hydromagnesite (Mg_5_(CO_3_)_4_(OH)_2_·4H_2_O, abbreviated as HM), also known as magnesium carbonate tetrahydrate, is a naturally abundant carbonate mineral found worldwide [20,21]. It boasts significant proven reserves, high purity, low cost, and ease of extraction. Our previous work proposed for the first time the utilization of HM in the formulation of dry powder fire-extinguishing agents [22]. Experimental investigations in liquid fire suppression have demonstrated its effective cooling and fire-extinguishing capabilities. However, the agent exhibits limitations in chemical efficacy, hindering its further popularization and application. In comparison to liquid fires, solid fires generally release more heat, burn more vigorously, and are more susceptible to smoldering, thereby posing greater challenges for extinguishment [23]. It has been reported that the P-O groups generated during the pyrolysis of diammonium hydrogen phosphate ((NH_4_)_2_HPO_4_, known as DAP) can effectively trap combustion radicals, thereby exhibiting a chemical fire-extinguishing effect [24,25]. Furthermore, DAP exhibits weak alkalinity, making its incorporation into alkaline HM feasible. Combining multiple components in dry powder fire-extinguishing agents is anticipated to introduce a synergistic effect and enhance the fire-extinguishing efficiency, thereby optimizing performance [26].

In this work, to address the limitation of HM’s effectiveness in liquid fires while exhibiting poor performance in solid fires, which restricts the broader application of HM dry powder, a novel composite dry powder fire-extinguishing agent was developed. Based on abundantly available HM materials, DAP was introduced to the composite dry powder through a physical blending modification process. The optimal proportion of DAP was determined by analyzing the dry powder consumption and extinguishing times obtained from the liquid fuel and solid fire-suppression experiments. MAP, DAP, and HM dry powder served as the control groups for comparison with the experimental HM/DAP dry powder in terms of fire-suppression performance. The physicochemical properties of the above dry powder were comprehensively investigated using scanning electron microscopy (SEM), X-ray diffraction (XRD), Brunauer–Emmett–Teller (BET) surface area analysis, and comprehensive performance assessment. The fire-extinguishing mechanism of the composite dry powder was elucidated through simultaneous thermal analysis (TG-DSC). The actual fire-suppression tests and the CO concentration measurements demonstrated that the HM/DAP composite dry powder exhibited superior fire-extinguishing performance and environmental friendliness, providing an effective solution for combating both liquid fuel and solid fires.

## 2. Experiments and Methods

### 2.1. Materials and Preparation Procedures

The HM (≥96% purity, supplied by Jiangsu Xibeli New Material Co., Ltd., Changzhou, China), DAP, and hydrophobic nano-silica (supplied by Shanghai Macklin Biochemical Technology Co., Ltd., Shanghai, China) were utilized directly without further purification or modification. A planetary ball mill (F-P4000, Hunan Focucy Experimental Instrument Co., Ltd., Changsha, China) was utilized for powder preparation. An efficient and uniform mixing between materials was achieved using a high-speed mixer (SHR-10A, Zhangjiagang City Hongji Machinery Co., Ltd., Zhangjiagang, China). The particle screening and grading were performed using a vibrating sieve particle size meter (FT-6300, Ningbo Rooko Instrument Co., Ltd., Ningbo, China).

The preparation process of the HM dry powder involved three main steps. Initially, the HM particles smaller than 45 μm in diameter were selected using a high-frequency vibration sieve for 10 min. Subsequently, 40 g of these selected powders were mixed with 5 wt.% of hydrophobic nano-silica for ball milling. The ratio of the grinding balls to the refining material was maintained at 5:1 in a planetary ball mill operating at 320 rpm for 40 min. Similarly, this procedure was repeated to produce the MAP and DAP dry powders for the blank control group.

The HM/DAP powders were prepared using a physical blending method, involving three sequential steps. Initially, 1000 g of sieved HM was combined with 5 wt.%, 10 wt.%, 15 wt.%, and 20 wt.% of DAP in a high-speed mixer for 15 min to produce HM/DAP particles. Subsequently, the mixed particles underwent a second sieving process through a 45 μm mesh sieve. Finally, 40 g of the second-sieved dry powder particles were weighed and mixed with 5 wt.% hydrophobic nano-silica for ball milling. The grinding ball to material ratio was maintained at 5:1 in a planetary ball mill set at 320 rpm for 30 min. The resulting HM/DAP dry powders were obtained following the completion of these procedures.

### 2.2. Microscopic Characterization Testing

Various analytical techniques were employed for the comprehensive characterization of the dry powder samples. The particle size and distribution were determined using a laser particle size analyzer (LS-909, Zhuhai OMEC Instruments Co., Ltd., Zhuhai, China). High-precision images of the powder’s microscopic surface structure were generated using a scanning electron microscope (SU3800, Hitachi Co., Ltd., Tokyo, Japan). The surface area, pore size, and pore volume distribution were assessed via nitrogen adsorption under vacuum conditions using an automatic specific surface and pore size analyzer (ASAP 2460, Micromeritics Co., Ltd., Norcross, GA, USA). The X-ray diffraction patterns were analyzed using an X-ray diffractometer (SmartLab, Rigaku Co., Ltd., Tokyo, Japan) with a scanning angle set at 10–60° and a scanning speed of 10°/min. The thermal properties, including heat loss, the thermal decomposition rate, and specific heat absorption/release, were evaluated using a simultaneous thermal analyzer (STA 449 F3, Netzsch Co., Ltd., Selb, Germany) under nitrogen atmosphere, with the heating parameters set at an initial temperature of 30 °C, a termination temperature of 800 °C, and a heating rate of 10 °C/min. The contact angle of the pressed powder samples (200 mg, 10 MPa) was measured using an automated contact angle measuring instrument (OCA20, Dataphysics, Ins, Co., Ltd., Filderstadt, Germany). Furthermore, parameters such as bulk density, vibration density, and angle of repose were analyzed using an automatic powder characteristic analyzer (FT-2000A, Ningbo Rooko Instrument Co., Ltd., Ningbo, China).

### 2.3. Experimental Apparatus

**Figure 1:**
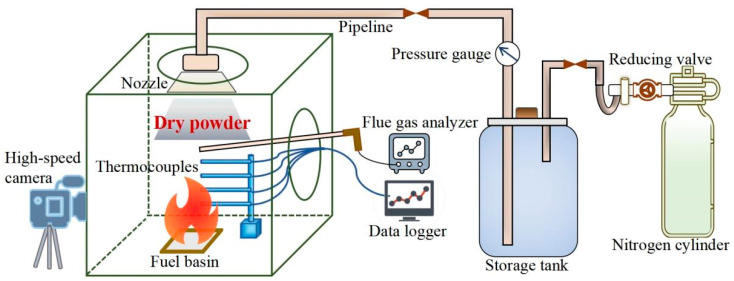
Schematic diagram of the experimental apparatus.

The fire-extinguishment experimental setup comprised a dry powder release apparatus, a 1 m^3^ small-scale fire-extinguishment test platform, and a data acquisition system (Figure 1). The dry powder release apparatus included nitrogen bottles, pressure-reducing valves, piping, pressure storage tanks, and nozzles. The 1 m^3^ laboratory platform featured five stainless steel plates and a high-temperature-resistant acrylic plate for observing flame morphology changes. The data acquisition system encompassed a temperature collector, a smoke analyzer, and a high-speed camera. To simulate realistic flame conditions and facilitate natural ventilation, the platform included a 200 mm diameter ventilation hole on both its top and its side. A 30 mm diameter nozzle positioned at the top ventilation hole released dry powder, and a 200 mm × 200 mm × 40 mm square fuel basin at the bottom of the platform contained n-heptane to simulate liquid fire. The solid fires were simulated by igniting a wood stack placed above the n-heptane-filled fuel basin. The wood stack device consisted of a stainless steel tube support measuring 500 mm × 500 mm × 100 mm, two steel pipes spaced 130 mm apart to support the solid strips, and 16 strips of pine solid sized 15 mm × 15 mm × 150 mm. Four K-type thermocouples with a 4 mm diameter and spaced 100 mm apart above the fuel basin were used to record the temperature variations of the flame root, the outer flame, the middle flame, and the inner flame. These temperature data were recorded by a temperature collector (LR8450, HIOKI (Shanghai) Measurement Technologies Co., Ltd., Shanghai, China) connected to the thermocouples. The dry powder consumption was determined by measuring the mass of the powder before and after the extinguishment using an electronic balance. A smoke probe, connected to the smoke analyzer (Testo 350, Testo Co., Ltd., Titisee-Neustadt, Germany), was positioned in the side vent of the fire-extinguishing experimental platform to record the smoke concentration data at the fire scene. Additionally, a high-speed camera (HDR-CX680, Sony Co., Ltd., Tokyo, Japan) was employed to capture the changes in the flame patterns and measure the fire-extinguishing time.

### 2.4. Experimental Methods for Liquid Fuel and Solid Fires

The liquid fuel and solid fire-extinguishing experiments were conducted using the HM/DAP dry powder for the experimental group, and three separate control groups were tested with the MAP dry powder, the DAP dry powder, and the HM dry powder. The experiments were performed under three different driving pressures: 0.1 MPa, 0.2 MPa, and 0.3 MPa. Each experiment utilized 50 g of the dry powder samples loaded into the powder storage tank. For the liquid fire experiments, 70 mL of n-heptane and 80 mL of deionized water were mixed in the square fuel basin. After igniting the n-heptane, the dry powder was released following approximately 30 s of combustion. The release of the dry powder ceased upon the extinguishing of the flame. In the solid fire experiments, 30 mL of n-heptane was used to ignite the wood stacks, followed by the release of the dry powder approximately 30 s later. The release of the dry powder ceased upon the extinguishing of the solid stack fire. To ensure the precision and reliability of the experimental results, each fire-extinguishing experiment was replicated three times.

## 3. Results and Discussion

### 3.1. Material Characterization

**Figure 2:**
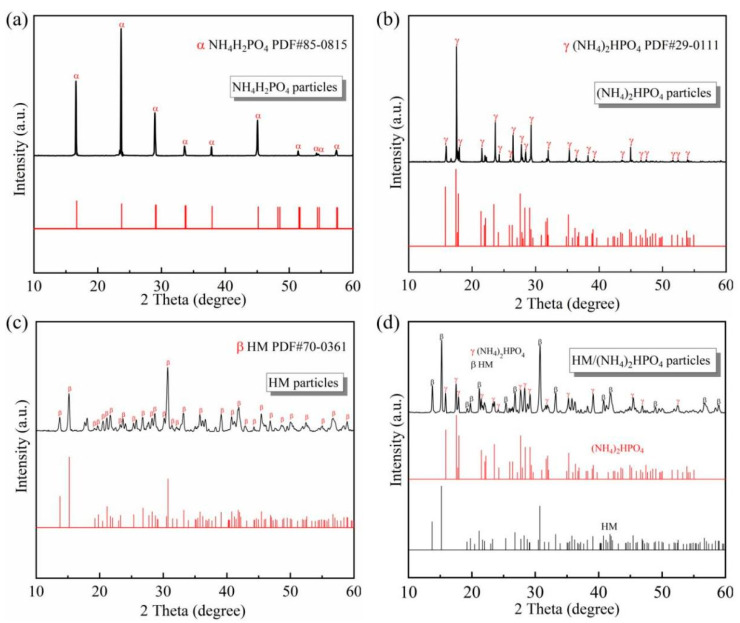
X-ray diffraction patterns of the fire-extinguishing agents. The red curves and black lines indicate the standard peaks and experimental test results of the powder, respectively.

Different chemical compositions influence the thermochemical decomposition mechanisms of dry powders during the fire-extinguishing process, leading to variations in their efficacy [27]. The crystal compositions of the as-prepared dry powder samples were analyzed by X-ray diffraction tests (Figure 2), with the key peaks compared against standard reference cards. The particles of MAP (Figure 2a) and DAP (Figure 2b) were identified as phosphates, while the HM particles (Figure 2c) consisted of alkali metal salts. The XRD pattern of the composite HM/DAP particles (Figure 2d) with 10 wt.% DAP exhibited key peaks characteristic of both HM and DAP, confirming the physical mixture of these two powders.

**Figure 3:**
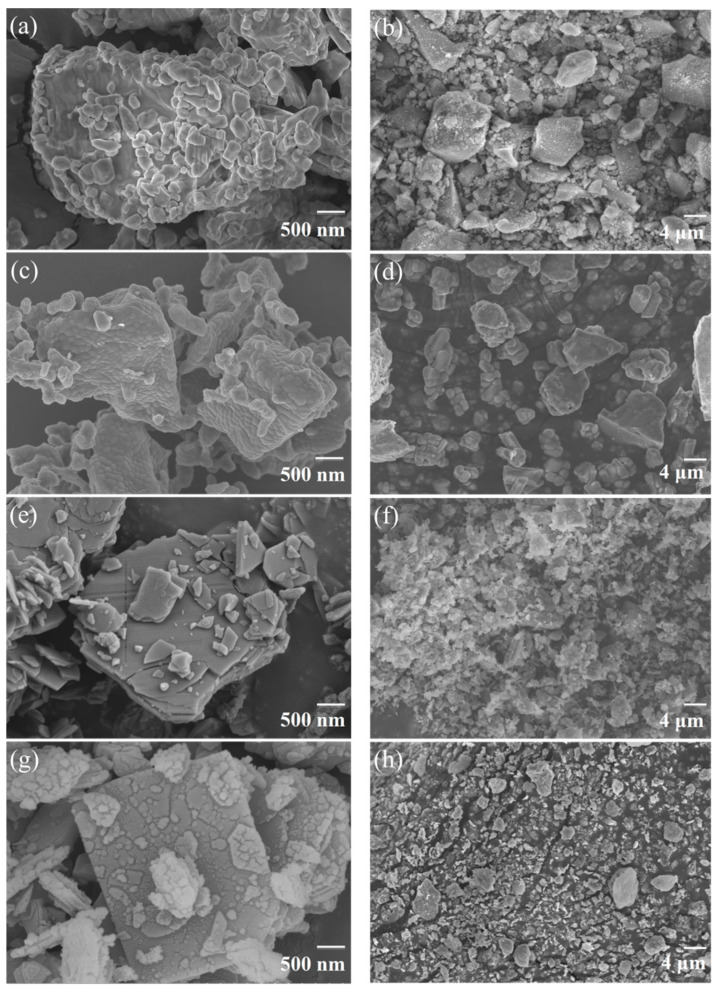
SEM images of the four types of dry powder particles: (**a**) MAP, (**b**) modified MAP, (**c**) DAP, (**d**) modified DAP, (**e**) HM, (**f**) modified HM, (**g**) HM/DAP, and (**h**) modified HM/DAP.

The microscopic morphology of dry powder particles significantly influences their fire-suppressing efficiency [22,28], with particle shape impacting the specific surface area and structural homogeneity promoting even dispersion and effective flame coverage. The figures in the left column (a, c, e, g) of Figure 3 show the microscopic morphology of four types of dry powder particles, while those in the right column (b, d, f, h) are their modified counterparts by hydrophobic nano-silica. The MAP particles (Figure 3a) exhibited a lumpy structure with a large particle size, dense small particles adhering to the surface, and a smooth particle surface. The DAP particles (Figure 3c) appeared irregularly lumpy with a moderate particle size, a rough surface, and a sparse distribution of small particles. The HM particles (Figure 3e) featured a plate-like structure with a polyhedral overall shape, smooth particle surfaces, and occasional small particle attachments. The HM/DAP particles with 10 wt.% DAP (Figure 3g) displayed a roughened surface with small particle attachments, maintaining a plate-like structure that enhanced the uniformity and consistency. The addition of hydrophobic silica further enhanced the uniformity of the dry powder, suggesting its superior performance on fire suppression.

**Figure 4:**
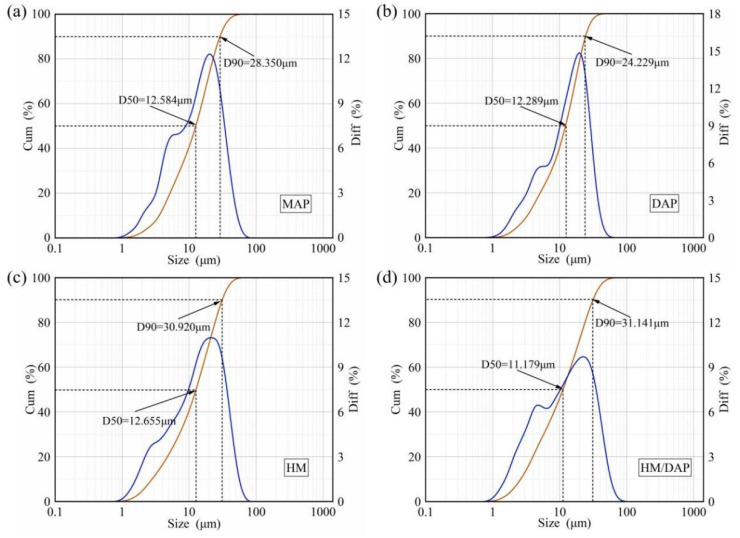
Particle size distribution of (**a**) MAP, (**b**) DAP, (**c**) HM, and (**d**) HM/DAP. The blue and brown curves represent particle size distribution of the dry powder and its integral, respectively.

In addition to particle shape, particle size significantly influences the effectiveness of dry powder in terms of flame coverage, penetration, and interaction with the flames in fire scenarios [29,30,31]. Figure 4 illustrates the particle size distribution of the four dry powder samples after mixing with the hydrophobic nano-silica. The analysis revealed that the median particle size (D50) of these samples ranged between ~11 μm and ~13 μm, which indicates a slight variation in the particle dimensions. Moreover, 90% of the dry powders (D90) exhibited particle sizes within the range of 24~32 μm, demonstrating a relatively narrow size distribution. Specifically, the HM/DAP dry powders with 10 wt.% DAP had D90 values at 34.141 μm, which exceeded those of the HM dry powders (30.920 μm), the MAP dry powders (28.350 μm), and the DAP dry powders (24.229 μm).

**Figure 5:**
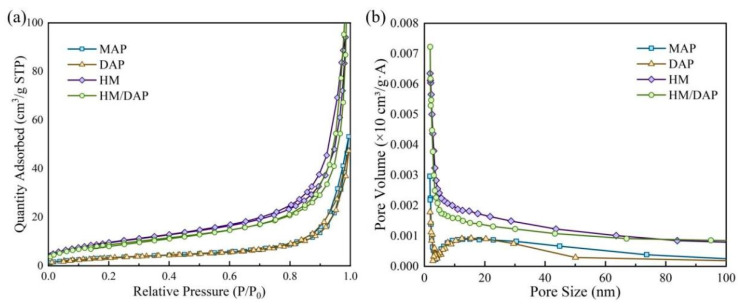
(**a**) Adsorption and desorption curves of the four samples, (**b**) Pore size distribution of the four samples.

During fire extinguishment, the specific surface area of dry powder extinguishants may play a crucial role in the adsorption of flame radicals, facilitating rapid diffusion and flame coverage and lowering the fire temperature. The adsorption–desorption isotherms and pore size distributions of the four dry powder samples are shown in Figure 5, and Table 1 presents the specific BET data. Both the HM dry powder and the HM/DAP dry powder exhibited superior specific surface area, pore volume, and pore size compared to the MAP and DAP dry powders. The specific surface area of the HM/DAP dry powder (32.834 m^2^/g) was slightly less than that of the HM dry powder (35.885 m^2^/g) but surpassed that of the MAP dry powder (12.800 m^2^/g) and the DAP dry powder (12.701 m^2^/g), implying the promising surface characteristics of the HM/DAP dry powder. Regarding pore volume, the HM/DAP dry powder (0.181 cm^3^/g) stood out as optimal among the four samples. Following the DAP modification, the pore diameter of the HM/DAP dry powder (18.31 nm) was marginally larger than that of the HM dry powder (16.64 nm). Overall, the addition of a small amount of DAP, which could contribute greatly to the chemical suppression effect, had a negligible impact on the surface area of the composite dry powder.

In practical applications, flowability significantly influences the dispersion of dry powder particles, consequently impacting the effectiveness of fire suppression [32]. Additionally, the hydrophobicity of dry powder extinguishing agents plays a critical role in maintaining particle dryness, preventing moisture absorption, and mitigating agglomeration issues [12,33]. Therefore, before conducting the fire-extinguishing experiments, the fluidity and hydrophobicity of the four dry powder samples were evaluated. Parameters such as loose packing density and vibration density are critical for assessing dry powder fluidity, with a smaller difference between these densities indicating superior fluidity [34]. The modified HM/DAP dry powders exhibited a reduced difference (0.02 g/cm^3^) compared to the HM dry powders (Table 2), suggesting enhanced fluidity. Furthermore, the flowability and angle of repose tests demonstrated that the HM/DAP dry powder achieved a flow rate comparable to that of the MAP and DAP dry powders (both 0.04 g/s), with an angle of repose (38°) lower than that of the HM dry powder (40°), which indicates an obvious improvement in the flowability of the composite dry powder [35,36]. The contact angle measurement indicated that the HM/DAP dry powder exhibited the highest hydrophobicity among the samples tested (90.54°), ensuring superior particle dryness and anti-caking properties compared to the other three dry powders.

### 3.2. Fire-Extinguishing Performance

#### 3.2.1. Liquid Fire Experiment

**Figure 6:**
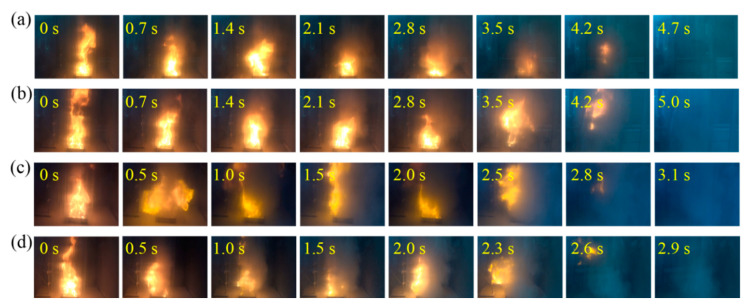
Images of the liquid-fire-extinguishing process of (**a**) MAP, (**b**) DAP, (**c**) HM, (**d**) HM/DAP.

Liquid-fire-extinguishing experiments were conducted for the four samples of MAP, DAP, HM, and composite dry powder with 10 wt.% DAP at driving pressures of 0.1 MPa, 0.2 MPa, and 0.3 MPa. Table 3 lists the extinguishing times and dry powder consumption of the four dry powder samples. Their corresponding flame changes at 0.2 MPa are shown in Figure 6. At this pressure, the difference in the extinguishing time between the HM/DAP dry powder (2.9 s) and the HM dry powder (3.1 s) was not significant. However, the amount of HM/DAP dry powder used (15.82 g) was 8.52 g less than that of the HM dry powder (24.34 g). The MAP dry powder and the DAP dry powder exhibited longer extinguishing times compared to the HM dry powder. At a lower pressure (0.1 MPa), the MAP dry powder (22.8 s) and the DAP dry powder (23.3 s) required more time to extinguish the flames compared to the HM and HM/DAP dry powders. The influence of the driving pressure on the HM and HM/DAP dry powders was minimal, whereas the MAP and DAP dry powders displayed more pronounced changes in extinguishing time and powder consumption with varying pressure. The effective extinguishing capability of the MAP and DAP dry powders relied on higher driving pressures, necessitating storage containers with greater pressure resistance.

In the existing research on extinguishing n-heptane fires, it is challenging to find studies conducted under identical experimental conditions, such as the n-heptane dosage, the spraying time of the extinguishing agents, and the other parameters. This variability complicates the direct comparison of the extinguishing effectiveness across the different studies. As shown in Table 3, for fire extinguishers utilizing MAP dry water [37] and ultrafine NaHCO_3_ dry powder [38], the former used a greater amount of n-heptane and required a longer extinguishing time compared to the present study. In contrast, the latter conducted experiments in a 0.1 m^3^ space with an unspecified amount of n-heptane, resulting in an extinguishing time of 1.29 s under the same pressure (0.2 MPa), which was shorter than the 2.9 s observed with the HM/DAP formulation. Nonetheless, it can be speculated that the HM/DAP dry powder will demonstrate superior fire-extinguishing performance under higher pressures or with smaller amounts of n-heptane. Overall, the modified HM/DAP dry powder demonstrated superior performance in both extinguishing time and dry powder consumption, establishing it as the most effective formulation among the four samples in this work.

**Figure 7:**
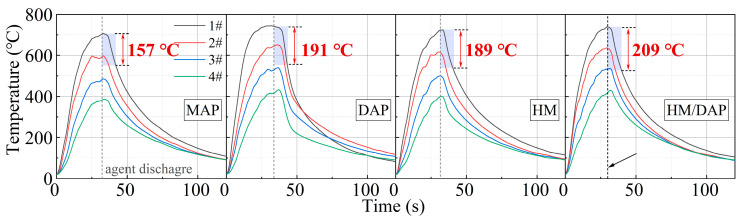
Change in temperature over time of MAP, DAP, HM, and HM/DAP for extinguishing liquid fires.

One possible reason for the superior fire-extinguishing effectiveness demonstrated by the composite dry powder in the temperature measurements at the fire scene is its optimal cooling capability. Figure 7 illustrates the temperature change curve of the fire under a 0.2 MPa driving pressure, with the shaded area representing the temperature reduction within 10 s after the release of the dry powder. Specifically, the sequence of temperature reduction within 10 s is as follows: HM/DAP (209 °C) > DAP (191 °C) > HM (189 °C) > MAP (157 °C). This comparison establishes the superior cooling effectiveness of the HM/DAP dry powder over the other three types of dry powder.

**Figure 8:**
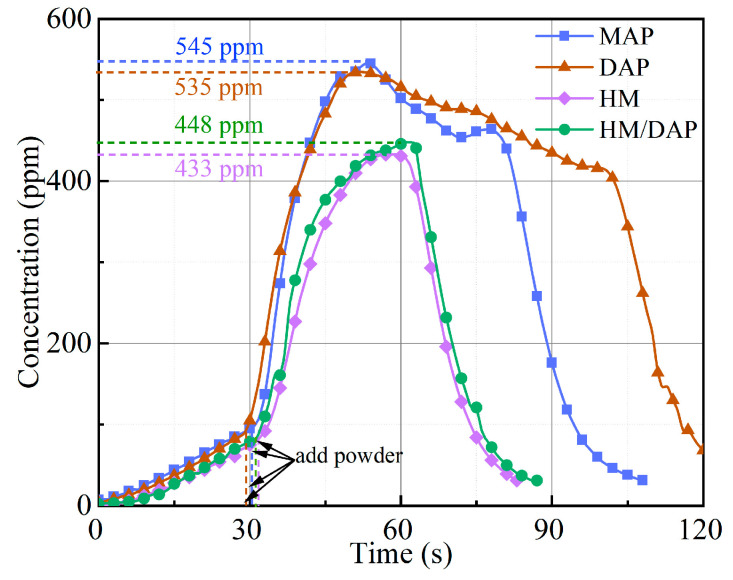
The change of CO concentration of the four powders for extinguishing liquid fires under 0.2 MPa.

Flame combustion releases toxic and harmful fumes that pose serious health risks to humans, with carbon monoxide (CO) from incomplete fuel combustion being particularly hazardous, capable of causing asphyxiation or death in severe cases. Therefore, evaluating the CO concentration of the four types of dry powder after fire suppression was essential. Before the release of the dry powder, the CO concentration measured approximately 80 ppm, and the highest post-release CO concentration values are shown in Figure 8. In a confined 1 m^3^ space, the introduction of the dry powder into a fire inhibited the combustion of the fuel and exacerbated the incomplete combustion of the flames, resulting in elevated CO concentrations. After reaching a peak, the CO concentration began to decrease, with varying degrees of inhibition observed among the four types of dry powder. The discharge of the HM/DAP and HM dry powders resulted in a rapid reduction in the CO concentration, demonstrating superior CO suppression capabilities. Conversely, the MAP and DAP dry powders exhibited inferior CO inhibition effects. These observations highlight that while the modified HM/DAP dry powder showed a slightly lower CO inhibition capability compared to the HM dry powder when extinguishing liquid fires, it still significantly outperformed the MAP and DAP dry powders in reducing CO levels. Notably, the introduction of the DAP substance led to a significant improvement of the fire-extinguishing efficiency, although the CO inhibition capability of the dry powder was slightly decreased.

#### 3.2.2. Solid Fire Experiment

In this study, the fire-extinguishing experiments were performed using HM/DAP dry powders with DAP mass fractions of 5 wt.%, 10 wt.%, 15 wt.%, and 20 wt.%. The optimal DAP addition was determined by evaluating the extinguishing time and dry powder consumption of the four different composite powders at a driving pressure of 0.2 MPa (Table 4). The 10 wt.% DAP/HM dry powder achieved the shortest extinguishing time (1.2 s) and required the least amount of dry powder (15.10 g). Consequently, the 10 wt.% DAP/HM dry powder was selected for further experimental investigation in this work.

**Figure 9:**
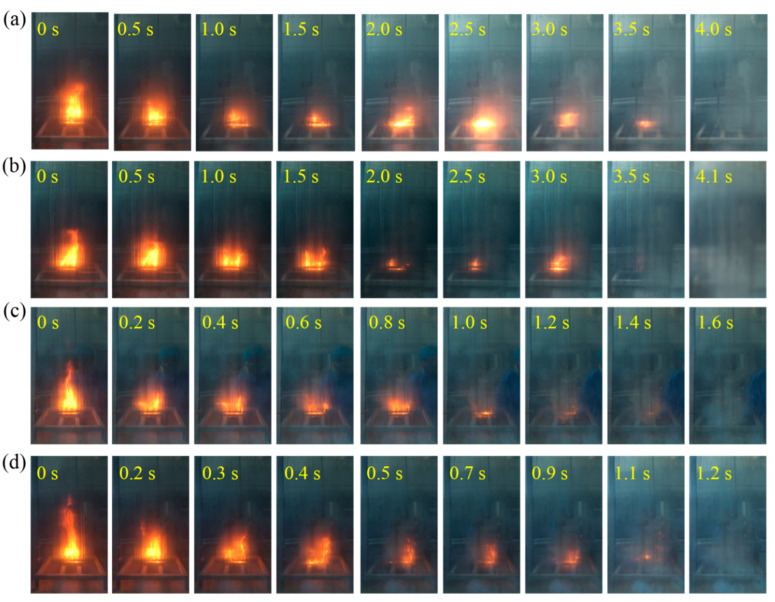
Images of the solid-fire-extinguishing process of (**a**) MAP, (**b**) DAP, (**c**) HM, (**d**) HM/DAP, for solid fires.

In this study, wood stacks measuring 150 mm × 150 mm × 60 mm were constructed using 16 pine wood strips. Table 5 lists the extinguishing time and dry powder consumption to extinguish the wood stack fires for the four dry powder samples at three sets of driving pressures: 0.1, 0.2, and 0.3 MPa. The alterations in the flame morphology subsequent to the discharge of the four dry powder samples under a driving pressure of 0.2 MPa are illustrated in Figure 9. Specifically, at 0.2 MPa, as evidenced by Table 5 and Figure 9, the HM/DAP dry powder exhibited a superior fire-extinguishing performance, achieving an extinguishing time of only 1.2 s, notably quicker compared to the MAP dry powder (4.0 s) and the DAP dry powder (4.1 s). Moreover, the HM/DAP dry powder required the least amount of dry powder (15.10 g) to extinguish the solid stack fire among the four samples. Table 5 also presents the reported results [39] for extinguishing wood stack (200 mm × 200 mm × 125 mm) fires, where both the commercial ABC dry powder and the MAP/Mg(OH)_2_ dry powder failed to suppress the fire at a pressure of 0.2 MPa. The enhanced extinguishing performance of the modified HM shows significant potential as an alternative to the MAP-based commercial dry powders. The practical applications in environments such as buildings, libraries, and laboratories, where MAP agents are currently employed, require further investigation and validation through additional research.

**Figure 10:**
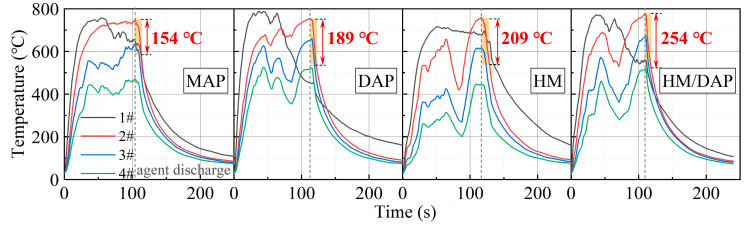
Change in temperature over time of MAP, DAP, HM, and HM/DAP, for solid fires.

Due to their distinct physicochemical properties, these powders exhibited varying trends in temperature reduction. The shaded area in Figure 10 represents the temperature decrease over 10 s following the release of the dry powders. The temperature decrease curve of the HM/DAP dry powder displays the steepest slope, achieving the largest temperature reduction. Specifically, the temperature decrease ranks as follows: HM/DAP (254 °C) > HM (209 °C) > DAP (189 °C) > MAP (154 °C), indicating the exceptional cooling efficacy of the HM/DAP dry powder.

**Figure 11:**
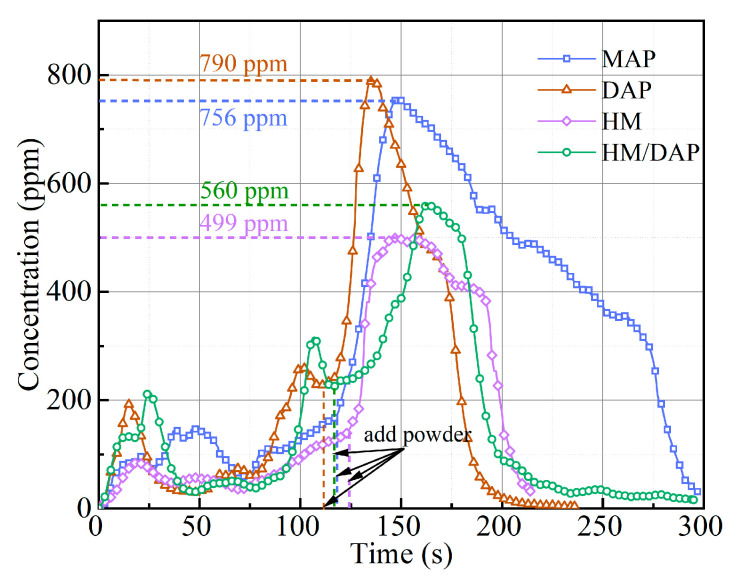
The change of CO concentration of the four powders for extinguishing solid fires under 0.2 MPa.

As illustrated in Figure 11, significant differences also exist in the inhibitory effects of the four dry powders on CO concentration during the extinguishment of solid fires. Specifically, the peak CO concentrations observed following the application of the HM dry powder (499 ppm) and the HM/DAP dry powder (560 ppm) were significantly lower than those recorded for the MAP dry powder (756 ppm) and the DAP dry powder (790 ppm). The marginally higher CO concentration associated with the HM/DAP dry powder compared to the HM dry powder is attributed to the chemical extinguishing action of DAP. This mechanism enhances flame suppression while also inducing incomplete combustion of the flame to a certain degree.

### 3.3. Fire-Extinguishing Mechanism Analysis

**Figure 12:**
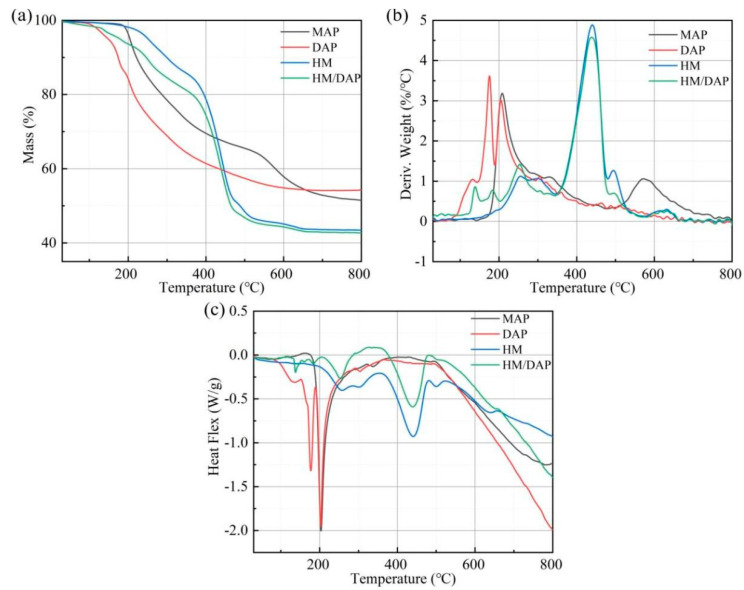
(**a**) Thermogravimetric (TG) curves, (**b**) Derivative thermogravimetric (DTG) curves, and (**c**) Differential scanning calorimetry (DSC) curves of the four samples.

The fire-suppression mechanism of composite dry powder is elucidated through simultaneous thermal analysis data (Figure 12). The thermogravimetric curve (Figure 12a) and previous studies [40,41] indicate that the HM dry powder decomposes at approximately 200 °C, releasing crystallization water between ~200 and ~350 °C, followed by water vapor and carbon dioxide between ~350 and ~650 °C, effectively diluting the oxygen. Its final product of pyrolysis is magnesium oxide [42,43], forming a dense oxide film on combustibles with an effective asphyxiating effect. The thermal decomposition rate curves (Figure 12b) show two peaks near 250 °C and 440 °C, indicating the HM dry powder’s robust thermal stability. The differential scanning calorimetry (Figure 12c) illustrates the HM dry powder’s endothermic pyrolysis process, which contributes to fire temperature reduction through heat absorption.

The extinguishing mechanisms of commercial MAP and DAP dry powder extinguishing agents have been reported, focusing on several aspects: the pyrolysis of the dry powder to lower the fire temperature, the release of intermediate products to reduce the oxygen concentration, the release of chemicals to interact with the combustion radicals, and the deposition of the pyrolysis end-products on combustible surfaces to prevent oxygen access. For instance, MAP dry powder undergoes pyrolysis releasing water vapor, ammonia, and phosphorus–oxygen groups that capture combustion radicals and form a dense oxidative film of phosphorus pentoxide on combustible surfaces, employing both physical and chemical mechanisms in fire suppression [44,45,46].

**Figure 13:**
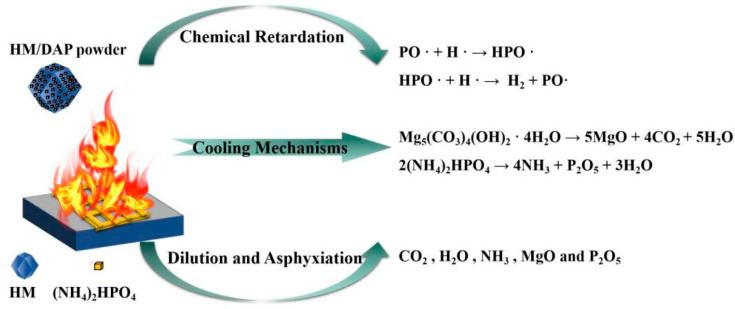
The fire-extinguishing mechanism of the HM/DAP dry powder.

As previously noted, the HM dry powder predominantly utilizes physical mechanisms such as cooling, dilution, and suffocation to suppress fires, thereby constraining its effectiveness. In contrast, the HM/DAP dry powder combines both physical and chemical inhibition mechanisms (Figure 13). DAP initiates pyrolysis at around 120 °C, releasing ammonia and water vapor from 120 to 550 °C, and forming phosphorus pentoxide and water from 550 to 750 °C [20,21]. The incorporation of DAP significantly enhances the fire-extinguishing capabilities of composite powders through two primary mechanisms: the introduction of chemical extinguishing effects and the extension of the thermal decomposition range of the dry powders. Specifically, DAP improves the fire-suppression effectiveness of composite dry powder by facilitating the chemical inhibition of free radicals during pyrolysis. Free radicals, including O·, H·, and OH·, which facilitate the combustion reaction, were adsorbed and consumed through collisions with the pyrolysis products (NH_4_^+^ and ·HPO_4_) [25,47]. The disruption of the chain reaction, driven by the continuous cyclic regeneration of the phosphorus–oxygen radicals during combustion, has been reported to significantly inhibit the combustion process. The extended thermal decomposition of the dry powder allows it to penetrate more deeply into the flame, promoting further decomposition and thereby achieving a more effective coverage. In summary, the HM/DAP composite dry powder exhibited a dual inhibitory effect, providing chemical inhibition at the flame surface and physical inhibition within the flame core.

In contrast to liquid fires, solid fires present a three-dimensional combustion structure, imposing greater demands on the efficacy of dry powder extinguishing agents. In the presence of sufficient oxygen, the residual charcoal within a burning wood pile may reignite; thus, it is crucial to enhance the coverage and isolation capabilities of the fire-extinguishing agent. The DAP/HM composite dry powder successfully achieved a dual suppression effect, combining chemical suppression at the flame surface with physical suppression at the flame root, thereby markedly improving its effectiveness in extinguishing solid fires.

## 4. Conclusions

This study investigated the fire-suppression efficacy of HM/DAP dry powder blends prepared via physical blending methods against both solid and liquid fires. The extinguishing performance of the blended dry powder was comprehensively assessed, yielding the following key findings:

(1) The scanning electron microscopy analysis revealed that blending DAP optimized the microstructure of the HM/DAP dry powder, facilitating a uniform diffusion and coverage on combustible surfaces upon deployment in fires.
(2) The fire-extinguishing experiments demonstrated that the modified HM/DAP dry powder displayed robust efficacy across different fire types (solid and liquid) and achieved an excellent extinguishing performance.
(3) The thermogravimetric analysis coupled with differential scanning calorimetry data indicated the prolonged pyrolysis of the HM/DAP dry powder, ensuring thorough contact with the flames. The introduction of DAP enabled the composite powder to realize the combination of chemical suppression on the flame surface and physical suppression at the flame base, thus enhancing fire suppression.
(4) The analysis of the CO concentrations at the fire scenes revealed that the application of the HM/DAP dry powder resulted in minimal CO emissions.

In summary, the physically modified HM/DAP dry powder demonstrated outstanding effectiveness in suppressing liquid fuel and solid fires. These findings offer valuable insights for the development of novel and efficient dry powder extinguishing agents in the field of fire-suppression technology.

## Figures and Tables

**Table 1:** Particle size and BET data of the four samples. S_BET_, V_Total_, and D represent specific surface area, pore volume, and pore size, respectively.

Powder	D50 (μm)	D90 (μm)	S_BET_ (m^2^/g)	V_Total_ (cm^3^/g)	D (nm)
MAP	12.584	28.350	12.800	0.058	19.15
DAP	12.289	24.229	12.701	0.073	21.52
HM	12.655	30.920	35.885	0.147	16.64
HM/DAP	11.179	31.141	32.834	0.181	18.31

**Table 2:** The all-around performance of the four samples.

Powder	Bulk Density (g/cm^3^)	Tap Density (g/cm^3^)	Fluidity (g/s)	Angle of Repose (°)	Contact Angle (°)
MAP	0.51	0.52	0.04	37	90.42
DAP	0.51	0.53	0.04	37	90.47
HM	0.47	0.50	0.03	40	90.10
HM/DAP	0.49	0.51	0.04	38	90.54

**Table 3:** Experimental and reported data on the effectiveness of liquid-fire-extinguishing powders.

Powder	Pressure (MPa)	Fire Extinction Time (s)	Powder Consumption (g)
MAP	0.1	22.8	27.42
	0.2	4.7	20.30
	0.3	2.1	14.57
MAP dry water ^1^	0.8	>40	16.88
Ultrafine NaHCO_3_^2^	0.2	1.29	6.09
DAP	0.1	23.3	24.15
	0.2	5.0	18.25
	0.3	2.7	13.74
HM	0.1	4.0	27.66
	0.2	3.1	24.34
	0.3	2.2	19.95
HM/DAP	0.1	4.7	23.18
	0.2	2.9	15.82
	0.3	2.0	12.65

^1^ MAP dry powder was utilized to extinguish a 100 mL n-heptane fire [37]. ^2^ An n-heptane fire was extinguished using NaHCO_3_ in a 0.1 m^3^ enclosed space, with the quantity of n-heptane remaining unspecified [38].

**Table 4:** Fire extinction time and agent mass consumption of samples with different masses of DAP under 0.2 MPa.

Powder	Fire Extinction Time (s)	Powder Consumption (g)
5 wt.% HM/DAP	1.4	18.67
10 wt.% HM/DAP	1.2	15.10
15 wt.% HM/DAP	1.6	18.89
20 wt.% HM/DAP	1.8	19.12

**Table 5:** Experimental data on the effectiveness of solid-fire-extinguishing powders.

Powder	Pressure (MPa)	Fire Extinction Time (s)	Powder Consumption (g)
MAP	0.1	12.7	26.22
	0.2	4.0	17.01
	0.3	2.2	12.57
C-ABC ^1^	0.2	Not extinguished	57
	0.6	4.6	39
MAP/Mg(OH)_2_^1^	0.2	Not extinguished	50
	0.6	3.4	30
DAP	0.1	14.2	22.82
	0.2	4.1	12.83 (smoldering)
	0.3	2.7	10.25
HM	0.1	1.8	25.05
	0.2	1.6	19.32
	0.3	1.2	17.15 (smoldering)
HM/DAP	0.1	1.9	23.71
	0.2	1.2	15.10
	0.3	0.9	10.68

^1^ The commercial ABC dry powder (C-ABC), containing 50 wt.% MAP, was reported to extinguish a wood stack (200 mm × 200 mm × 125 mm) fire in a 1 m^3^ chamber [39].

## Data Availability

The original contributions presented in this study are included in the article. Further inquiries can be directed to the corresponding authors.

## References

[1] Chowdhury R.B., Moore G.A., Weatherley A.J., Arora M. (2017). Key sustainability challenges for the global phosphorus resource, their implications for global food security, and options for mitigation. J. Clean. Prod..

[2] Reijnders L. (2014). Phosphorus resources, their depletion and conservation, a review. Resour. Conserv. Recycl..

[3] Ulrich A.E., Frossard E. (2014). On the history of a reoccurring concept: Phosphorus scarcity. Sci. Total Environ..

[4] Edixhoven J.D., Gupta J., Savenije H.H.G. (2014). Recent revisions of phosphate rock reserves and resources: A critique. Earth Syst. Dynam..

[5] Wu F., Wang J., Liu J., Zeng G., Xiang P., Hu P., Xiang W. (2021). Distribution, geology and development status of phosphate resources. Geol. China.

[6] Cheng M., Shi C., Hao L., Wang X., Guo X., Liu R., Hao X. (2023). Sustainable development of phosphorus recovery: From a product perspective. Sustain. Prod. Consum..

[7] Hu X., Wang J., Wu F., Li D., Yang J., Chen J., Liang J., Lou X., Chen H. (2023). Phosphorus recovery and resource utilization from phosphogypsum leachate via membrane-triggered adsorption and struvite crystallization approach. Chem. Eng. J..

[8] Zhu F., Cakmak E.K., Cetecioglu Z. (2023). Phosphorus recovery for circular Economy: Application potential of feasible resources and engineering processes in Europe. Chem. Eng. J..

[9] Zhu H., Li Z., Zhao J., Li R. (2024). Preparation of novel gel foam and its fire suppression performance against gasoline pool fires. Energy.

[10] Meng X., Wang Z., Liu B., Gao Y., Zhang J., Sun J., Wang Q. (2024). Enhancing extinguishing efficiency for lithium-ion battery fire: Investigating the extinguishing mechanism and surface/interfacial activity of F-500 microcapsule extinguishing agent. eTransportation.

[11] Kim S., Choi H., Song Y.K., Kim S., Noh S.M., Lee K.C. (2024). Thermoresponsive microcapsules with fire-extinguishing composites for fire prevention. ACS Appl. Polym. Mater..

[12] Zhou Y., Zhao J., Fu Y., Yu Z., Lu S., Zhang H., Jiang Y. (2024). Study on the hydrophobic nano silica particles as flow-enhancing additives for ultrafine dry powder fire extinguishing agent. Adv. Powder Technol..

[13] Isola M., Colucci G., Diana A., Sin A., Tonani A., Maurino V. (2024). Thermal properties and decomposition products of modified cotton fibers by TGA, DSC, and Py–GC/MS. Polym. Degrad. Stab..

[14] Pantaleoni A., Sarasini F., Russo P., Passaro J., Giorgini L., Bavasso I., Santarelli M.L., Petrucci E., Valentini F., Bracciale M.P. (2024). Facile and bioinspired approach from gallic acid for the synthesis of biobased flame retardant coatings of basalt fibers. ACS Omega.

[15] Cao F.-C., Ma X.-Y., Li Q.-R., Tang Y., Dong X.-L., Huang A.-C. (2024). Investigating the efficacy of expanded graphite/NaCl composite powder as an extinguishing agent for metal combustion suppression. J. Loss Prev. Process Ind..

[16] Zhang Y., Wang Z., Li Q., Pan R., Zhou X. (2024). A novel approach for enhancing fire suppression efficiency of dry powder extinguishant: From the synergistic effect of dawsonite. Powder Technol..

[17] Bifulco A., Climaco I., Casciello A., Passaro J., Battegazzore D., Nebbioso V., Russo P., Imparato C., Aronne A., Malucelli G. (2025). Prediction and validation of fire parameters for a self-extinguishing and smoke suppressant electrospun PVP-based multilayer material through machine learning models. J. Mater. Sci..

[18] Guo Y., Chang Z., Tan Z. (2023). Study on fire extinguishing mechanism of allophanate based on Monnex dry powder. Comput. Theor. Chem..

[19] Zhang Y., Wang Z., Liu J., Li Q., Pan R., Zhou X. (2023). Alkaline potassium aluminum carbonate: A novel high-efficiency dry powder extinguishing agent with high heat-resistant. J. Anal. Appl. Pyrolysis.

[20] Atay H.Y., Çelik E. (2010). Use of Turkish huntite/hydromagnesite mineral in plastic materials as a flame retardant. Polym. Composite..

[21] Power I.M., Wilson S., Thom J.M., Dipple G.M., Gabites J.E., Southam G. (2009). The hydromagnesite playas of Atlin, British Columbia, Canada: A biogeochemical model for CO_2_ sequestration. Chem. Geol..

[22] Wang M., Zhou Z., Liang Z., Du S., Cai G., Wang X., Zhou Y., Zhang H. (2024). The preparation and fire extinguishing mechanism research of a novel high-efficiency KHCO_3_@HM dry powder. Mater. Today Commun..

[23] Cai N., Chow W.-k. (2014). Numerical studies on heat release rate in a room fire burning wood and liquid fuel. Build. Simul..

[24] Hautamäki S., Altgen M., Altgen D., Larnøy E., Hänninen T., Rautkari L. (2020). The effect of diammonium phosphate and sodium silicate on the adhesion and fire properties of birch veneer. Holzforschung.

[25] Zhao Q., Chen X., Dai H., Huang C., Liu J., He S., Yuan B., Yang P., Zhu H., Liang G. (2020). Inhibition of diammonium phosphate on the wheat dust explosion. Powder Technol..

[26] Wang Q., Jiang X., Deng J., Luo Z., Wang Q., Shen Z., Shu C.-M., Peng B., Yu C. (2023). Analysis of the effectiveness of Mg(OH)_2_/NH_4_H_2_PO_4_ composite dry powder in suppressing methane explosion. Powder Technol..

[27] Wang Y.-Y., Zhu F.-H., Zhou H.-L., Chu S.-L., Jiang J.-C., Huang A.-C. (2024). Investigation in the fire suppression properties of KHCO_3_ and K_2_C_2_O_4_ dry water incorporates core-shell structures. J. Loss Prev. Process Ind..

[28] Chai G., Xie Y., Wang Y., Zhu G., Markert F. (2024). Experimental study on preparation of a new cleaner water-based powder agent and its extinguishing efficiency and thermodynamic mechanism on a typical fuel fire. J. Therm. Anal. Calorim..

[29] Du D., Shen X., Feng L., Hua M., Pan X. (2019). Efficiency characterization of fire extinguishing compound superfine powder containing Mg(OH)_2_. J. Loss Prev. Process Ind..

[30] Liang Z., Liu J., Wan Y., Feng Z., Zhang P., Wang M., Zhang H. (2023). Preparation and fire extinguishing mechanism of novel fire extinguishing powder based on recyclable struvite. Mater. Today Commun..

[31] Zhang L., Feng Y., Wu S., Jia H. (2024). Numerical study of the effect of primary nozzle geometry on supersonic gas-solid jet of bypass injected dry powder fire extinguishing device. Fire.

[32] Lu G., Zhao J., Zhou Y., Fu Y., Lu S., Zhang H. (2024). Study on flowability enhancement and performance testing of ultrafine dry powder fire extinguishing agents based on application requirements. Fire.

[33] Zhao J., Yin Z., Usman Shahid M., Xing H., Cheng X., Fu Y., Lu S. (2019). Superhydrophobic and oleophobic ultra-fine dry chemical agent with higher chemical activity and longer fire-protection. J. Hazard. Mater..

[34] Wang Z., Guo X., Liu J., Zhang Z., Pan X., Hua M., Wu Z., Jiang J. (2024). Experimental study on the inhibition of hydrogen deflagration by flame-retardant compounded ultrafine dry powder fire extinguishing media containing zinc hydroxystannate. Renew. Energy.

[35] Wang Y., Zhu G., Chai G., Zhou Y., Chen C., Zhang W. (2021). Experimental study on the effect of release pressure on the extinguishing efficiency of dry water. Case Stud. Therm. Eng..

[36] Zheng X., Kou Z., Liu S., Cai G., Wu P., Huang Y., Yang Z. (2023). Preparation and properties of a new core–shell-modified gel dry-water powder. Powder Technol..

[37] Han Z., Gong L., Du Z., Duan H. (2020). A Novel Environmental-Friendly Gel Dry-Water Extinguishant Containing Additives with Efficient Combustion Suppression Efficiency. Fire Technol..

[38] Zhang Y., Hou H., Wang Y., Pan R., Zhou X. (2024). Preparation of NaHCO_3_ fire extinguishing agent with perfluoropolyether groups to inhibit rapid re-ignition of Class B fires. Colloids Surf. Physicochem. Eng. Asp..

[39] Hangchen L., Xiaohui S., Xinxin G., Shunchao L., Han Z., Chendong Z., Min H., Xuhai P. (2021). High efficiency of the NH_4_H_2_PO_4_/Mg(OH)_2_ composite for guaranteeing safety of wood production. J. Loss Prev. Process Ind..

[40] Hollingbery L.A., Hull T.R. (2010). The thermal decomposition of huntite and hydromagnesite—A review. Thermochim. Acta.

[41] Hollingbery L.A., Hull T.R. (2012). The thermal decomposition of natural mixtures of huntite and hydromagnesite. Thermochim. Acta.

[42] Hollingbery L.A., Hull T.R. (2010). The fire retardant behaviour of huntite and hydromagnesite—A review. Polym. Degrad. Stab..

[43] Hollingbery L.A., Hull T.R. (2012). The fire retardant effects of huntite in natural mixtures with hydromagnesite. Polym. Degrad. Stab..

[44] Fan R., Jiang Y., Jiang H. (2021). Experimental and theoretical investigation of dry-water containing phosphoric acid for new fire suppressant. J. Loss Prev. Process Ind..

[45] Pardo A., Romero J., Ortiz E. (2017). High-temperature behaviour of ammonium dihydrogen phosphate. J. Phys. Conf. Ser..

[46] Zhang C., Li H., Guo X., Li S., Zhang H., Pan X., Hua M. (2021). Experimental and theoretical studies on the effect of Al(OH)_3_ on the fire-extinguishing performance of superfine ABC dry powder. Powder Technol..

[47] Wang J., Li H., Diao S., Yao Y., Wei C., Yu M. (2024). Reactive behaviors and mechanism of methane/coal dust hybrid explosions inhibited by NH_4_H_2_PO_4_ and (NH_4_)_2_HPO_4_: A combined ReaxFF-MD and DFT study. Chem. Eng. J..

